# Cost-effectiveness of a participatory return-to-work intervention for temporary agency workers and unemployed workers sick-listed due to musculoskeletal disorders: design of a randomised controlled trial

**DOI:** 10.1186/1471-2474-11-60

**Published:** 2010-03-28

**Authors:** Sylvia J Vermeulen, Johannes R Anema, Antonius JM Schellart, Willem van Mechelen, Allard J van der Beek

**Affiliations:** 1 Department of Public and Occupational Health, EMGO Institute for Health and Care Research, VU University Medical Centre, Amsterdam, The Netherlands; 2 Dutch Research Center for Insurance Medicine AMC-UWV-VUmc, VU University Medical Centre, Amsterdam, The Netherlands

## Abstract

**Background:**

Within the working population there is a vulnerable group: workers without an employment contract and workers with a flexible labour market arrangement, e.g. temporary agency workers. In most cases, when sick-listed, these workers have no workplace/employer to return to. Also, for these workers access to occupational health care is limited or even absent in many countries. For this vulnerable working population there is a need for tailor-made occupational health care, including the presence of an actual return-to-work perspective. Therefore, a participatory return-to-work program has been developed based on a successful return-to-work intervention for workers, sick-listed due to low back pain.

The objective of this paper is to describe the design of a randomised controlled trial to study the (cost-)effectiveness of this newly developed participatory return-to-work program adapted for temporary agency workers and unemployed workers, sick-listed due to musculoskeletal disorders, compared to usual care.

**Methods/Design:**

The design of this study is a randomised controlled trial with one year of follow-up. The study population consists of temporary agency workers and unemployed workers sick-listed between 2 and 8 weeks due to musculoskeletal disorders. The new return-to-work program is a stepwise program aimed at making a consensus-based return-to-work implementation plan with the possibility of a (therapeutic) workplace to return-to-work. Outcomes are measured at baseline, 3, 6, 9 and 12 months. The primary outcome measure is duration of the sickness benefit period after the first day of reporting sick. Secondary outcome measures are: time until first return-to-work, total number of days of sickness benefit during follow-up; functional status; intensity of musculoskeletal pain; pain coping; and attitude, social influence and self-efficacy determinants. Cost-benefit is evaluated from an insurer's perspective. A process evaluation is part of this study.

**Discussion:**

For sick-listed workers without an employment contract there can be gained a lot by improving occupational health care, including return-to-work guidance, and by minimising the 'labour market handicap' by creating a return-to-work perspective. In addition, reduction of sickness absence and work disability, i.e. a reduction of disability claims, may result in substantial benefits for the Dutch Social Security System.

**Trial registration:**

Trial registration number: NTR1047.

## Background

### Vulnerable working population

To date, most research regarding occupational health care and return-to-work (RTW) is aimed at sick-listed employees, i.e. workers with an employment contract, and the majority of developed occupational health care intervention programs is workplace-based or contain a workplace component [1-9]. However, within the working population there is a vulnerable group, namely workers without an employment contract and workers with flexible labour market arrangements, e.g. temporary agency workers. This vulnerable group consists of relatively younger persons, more (partly) occupationally disabled, and more immigrants. Furthermore, this group is characterised by a lower education, a lower socio-economic status, less job security, a greater distance to the labour market [10-13], and an increased risk for work disability[10,14,15].

In most cases, when sick-listed, these workers have no workplace/employer to return to[16,17]. Also, for these workers access to occupational health care is limited or even absent in many countries [18-20], and when available occupational health care and RTW guidance appears to be inadequate[11]. In addition, literature shows that work itself[21], creating a supportive work climate and, if necessary, (temporary) work(place) accommodations[22,23] are important factors in facilitating RTW. Therefore, adequate, i.e. tailor-made, occupational health care for this group of workers with the presence of a workplace for (therapeutic) RTW seems to be an important factor in the recovery and (vocational) rehabilitation process[16].

### The Dutch Social Security System

In the Netherlands the Sickness Benefit Act, carried out by the Social Security Agency (SSA), provides supportive income, i.e. sickness benefit, for workers without an employment contract who become sick-listed. After reporting sick, the worker is entitled to occupational health care by the SSA during his/her sickness benefit period. Vocational rehabilitation is carried out by a team of occupational health care professionals from the SSA, consisting of an insurance physician, a labour expert, and a case-manager. The insurance physician of the SSA guides the worker according to Dutch guidelines for occupational health care. In addition, there are general obligatory occupational health care interventions, as dictated by Dutch legislation, such as inviting to consulting hours, discussing and advising about RTW, and making of a RTW action plan. In principle, when the worker is 6 weeks sick-listed he/she is invited to visit the SSA for a medical assessment by the insurance physician. The aim of this first medical assessment is to certify sickness and thereby approving the sickness benefit claim, and a to make a (medical) problem analysis with advising about recovery, e.g. health promotion, and RTW options. The occupational health care by the SSA ends when the worker is no longer sick-listed and the sickness benefit ends. When the worker is still partially or fully work disabled after two years, he/she can apply for a long-term disability benefit. This is the same as for long-term sick-listed workers with an employment contract.

### A participatory RTW intervention

The structured and stepwise process of development, implementation and evaluation of a theory and practise-based participatory RTW program for temporary agency workers and unemployed workers, sick-listed due to musculoskeletal disorders (MSD) was recently published[16]. This intervention is based on the already developed and cost-effective RTW program for employees, sick-listed due to low back pain[24,25]. Intervention Mapping (IM) [26-28] was used to specifically tailor the new RTW program taking into account the target group, the users and the context in which the RTW program is implemented. The IM protocol strongly supported obtaining input from different stakeholders (i.e. sick-listed temporary agency workers, sick-listed unemployed workers, occupational health care professionals from the SSA, temporary agencies, and vocational rehabilitation agencies) to ensure participation and involvement in all steps of program development and implementation.

To enhance the success of future implementation, focus groups were held with stakeholders about important factors for innovations, such as potential advantage, complexity of the new program, and compatibility with daily practise[29]. This resulted in important keystones to be incorporated in the RTW program, namely: the presence of a RTW perspective (i.e. creating a (therapeutic) workplace), an independent RTW coordinator who guides the process to achieve consensus, the most suitable moment to apply the protocol, and a structural communication link between all stakeholders. The newly developed RTW program consists of a stepwise process to identify and solve obstacles for RTW by the sick-listed temporary agency worker or sick-listed unemployed worker and his/her labour expert from the SSA, resulting in a consensus-based implementation plan to facilitate (therapeutic) RTW. Since there is (in most cases) no workplace to return to, agreements were made with four vocational rehabilitation agencies to offer temporary (therapeutic) workplaces.

### Objective

The objective of this paper is to describe the design of a randomised controlled trial (RCT) to study the (cost-)effectiveness of this new participatory RTW program for temporary agency workers and unemployed workers, sick-listed due to MSD, compared to usual care.

## Methods/Design

To describe the design of the RCT, the CONSORT statement[30,31] was followed. The goal of this checklist is to improve the quality of reporting of randomised controlled trials.

### Organisation of the study

**Figure 1:**
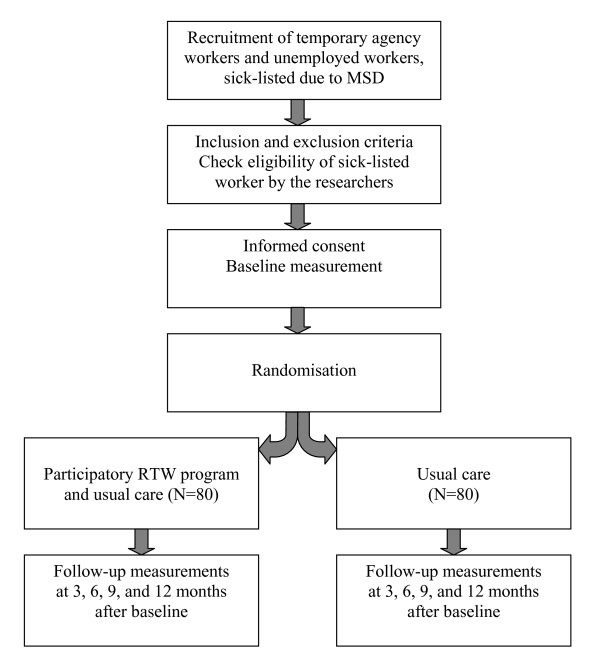
**Design of the randomised controlled trial**.

The study design is a randomised controlled trial with a follow-up of one year (see figure 1). An economic evaluation is conducted alongside the RCT. The RCT is conducted in collaboration with five front offices of the Social Security Agency (SSA) and four large Dutch vocational rehabilitation agencies (Olympia, Adeux, Capability, and Randstad Rentrée) in the eastern part of the Netherlands.

To monitor the conduct of the study, a project group is formed, consisting of the researchers, representatives of the SSA (e.g. staff, management and occupational health care professionals), and representatives of the participating vocational rehabilitation agencies. The most important task of this project group is to identify and solve barriers for implementation of the participatory RTW program and working with the program in daily practice. The Medical Ethics Committee of the VU University Medical Centre (Amsterdam, the Netherlands) approved the study design, the protocols and procedures, and informed consent. Towards the stakeholders and participants, the RCT is entitled the STEP-UP study.

### Study population

The population in this study consists of temporary agency workers and unemployed workers, who live in the eastern part of the Netherlands and when sick-listed come under one of the five following front offices of the Social Security Agency: UWV Arnhem, UWV Apeldoorn, UWV Hengelo, UWV Nijmegen, or UWV Zwolle. The main inclusion criteria are: 1. being a temporary agency worker or unemployed worker; 2. being between 18 and 64 years of age; 3. being sick-listed between 2 and 8 weeks; and 4. having MSD as main reason for a sickness benefit claim. The main exclusion criteria are: 1. an accepted sickness benefit claim and being sick-listed for more than 8 weeks; 2. not being able to complete questionnaires written in the Dutch language; 3. having a conflict with the SSA or the Dutch Institute for Benefit Schemes (UWV) regarding a sickness benefit claim or a long-term disability claim, respectively; 4. the presence of a legal conflict, e.g. an ongoing injury compensation claim; and 5. an episode of sickness absence due to MSD within one month before the current sickness benefit claim. After inclusion and randomisation the insurance physician of the SSA is asked to identify workers with severe co-morbidity; i.e. terminal disease, serious psychiatric disorders, or serious cardio-vascular disease, since these are contra-indications for receiving the participatory RTW program. These participants are prevented from starting with the participatory RTW program. However, following the intention-to-treat principle, they remain in the allocated study group (intervention or control). For an overview of all inclusion and exclusion criteria, see table 1.

**Table 1:** Overview of inclusion and exclusion criteria

Inclusion criteria
• temporary agency worker or unemployed worker
• age between 18 and 64 years
• sick-listed between 2 and 8 weeks
• MSD complaints as main reason for reporting sick
• able to complete questionnaires written in Dutch



**Exclusion criteria**

• sick-listed for more than 8 weeks
• not able to complete questionnaires written in Dutch
• a conflict with the SSA or UWV regarding a sickness benefit claim or a long term disability claim
• a legal conflict, e.g. an injury compensation claim
• episode of sickness absence due to MSD within one month before current sickness benefit claim
• revision or ending of a disability benefit within one month before current sickness benefit claim
• absence of work abilities due to medical reasons for at least three months
• serious physical disease, e.g. cancer
• serious psychiatric co-morbidity
• serious cardiovascular co-morbidity
• pregnancy until three months after delivery

### Recruitment of participants

For the recruitment of participants the database of the SSA is used. When reporting sick not only personal data, but also the reason for this, i.e. the health problem, is registered (using codes) in a computerised client record system. Based on a weekly query of this record system, all temporary agency workers and unemployed workers who are sick-listed between one and two weeks due to MSD, and live in the eastern part of the Netherlands receive a letter from the insurance physician of the SSA, on behalf of the researchers. The aim of this letter is to give information about the study and to ask for their participation. In addition, they also receive an information flyer with more details about the study, a screening questionnaire, and a return envelope for the screening questionnaire. The reason for approaching potential participants in the second week of sick leave is the time period in which a RTW action plan has to be made, i.e. 8 weeks after the first day of reporting sick. This is obligated according to the Dutch Improved Gatekeeper Act. Furthermore, it has been shown that early RTW intervention is important to prevent long-term work disability[4,32-34].

Temporary agency workers and unemployed workers who return the questionnaire, and meet the criteria (being temporary agency worker or unemployed worker, and still sick-listed), and indicate that they are willing to participate are contacted by the researchers by telephone. In this telephone call additional information is given about the content of study and the implications of participation. Using the formulated inclusion and exclusion criteria, the eligibility of the worker is checked. If the temporary agency worker or unemployed worker meets all selection criteria and still wants to participate, an intake appointment with the research assistant is planned at one of the UWV front offices. The worker receives a confirmation of this appointment by postal mail, including a detailed information brochure about the study. During the meeting with the research assistant the worker gives informed consent, fills in the baseline questionnaire, and randomisation is performed.

### The participatory RTW program

**Figure 2:**
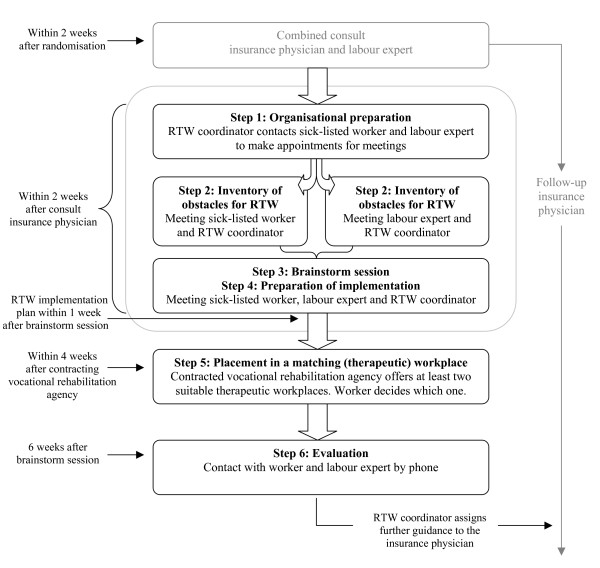
**Content of the participatory return-to-work program**.

The aim of the new RTW program is to make a consensus-based RTW implementation plan. The three main stakeholders in this intervention are: the sick-listed worker himself/herself, the labour expert of the SSA who guides the worker with regard to vocational rehabilitation, and an independent RTW coordinator. The program starts with identifying obstacles for RTW, followed by a brainstorm session in which the sick-listed worker and the labour expert formulate solutions/possibilities for suitable (therapeutic) work. This process results in the making of a consensus-based RTW implementation plan. The RTW coordinator has a key role[35], not in the role of RTW expert, but he/she has to stimulate active involvement of both the sick-listed worker and the labour expert during the whole process and guide them towards consensus. In this study the RTW coordinator is an employee of the SSA with good process guiding skills, an independent position, and sufficient knowledge and experience regarding (vocational) rehabilitation. To guarantee the independence of the RTW coordinator he/she has no other involvement regarding vocational rehabilitation of the sick-listed worker concerned. Furthermore, to create an actual RTW perspective, a vocational rehabilitation agency is contracted to find a (therapeutic) workplace matching with the formulated RTW implementation plan and taking into account the worker's (functional) limitations. For an overview of the steps of the new participatory RTW program and the stakeholders involved, see figure 2.

### Combined consult insurance physician and labour expert

All participants receive usual care by the insurance physician of the SSA, i.e. treatment/guidance according to Dutch guidelines for occupational health care. The participants in the intervention group receive a home assignment from the insurance physician in the first consult. They are asked to make an inventory of RTW obstacles, whether it be work or non-work related, as starting-point for the first meeting with the RTW coordinator. To prevent conflicting advice about RTW the insurance physician sends a letter with an information brochure about the participatory RTW program to the general practitioner of the sick-listed worker. To ensure that the labour expert has sufficient information regarding the sick-listed worker before the start of the RTW program, the sick-listed worker has a consult with the labour expert directly following the first consult with the insurance physician.

### Organisation and preparation

The RTW coordinator checks if the worker has had the combined consult with the insurance physician and the labour expert. Next, he/she contacts the worker and the labour expert by telephone to plan meetings for the inventory of obstacles and the brainstorm session. These meetings have to take place within two weeks after the consult with the insurance physician.

### Inventory of obstacles for RTW

The RTW coordinator explains to the sick-listed worker and the labour expert that the aim of the program is a consensus-based process to identify obstacles for RTW and to choose solutions, i.e. possibilities regarding type of work(place), work content and necessary preconditions (work or non-work related), to achieve RTW. Furthermore, the RTW coordinator explains that guiding the process with equal contribution by the sick-listed worker and the labour expert is his/her main goal.

In the meeting with the sick-listed worker the RTW-coordinator uses the inventory of obstacles for RTW (given to the worker in the first consult with the insurance physician) as a starting point. During the interview obstacles for RTW are identified. Next, these obstacles are prioritised based on frequency (how often do they occur?) and severity (how large is the impact on functioning in daily life and/or work?). Subsequently, the RTW coordinator has a meeting with the labour expert. The procedure is similar to the interview with the sick-listed worker and results in a selection of prioritised obstacles for RTW from the perspective of the labour expert. Finally, the RTW coordinator summarizes the results and formulates the prioritised barriers for RTW to be discussed in the brainstorm session.

### Brainstorm session

At the start of the brainstorm session the RTW coordinator explains the summary of prioritised obstacles for RTW consisting of the three main obstacles identified by the sick-listed worker and the three main obstacles identified by the labour expert. Next, the RTW coordinator explains the brainstorm procedure. Based on the nominal group technique[24] the sick-listed worker and the labour expert both have to think about solutions for all six prioritised obstacles. The proposed solutions are judged on the basis of availability, feasibility and ability to solve the barrier. The end goal of this session is to achieve consensus between the sick-listed worker and the labour expert about the most suitable and feasible solutions.

### Preparation of implementation

Together, the sick-listed worker, the labour expert, and the RTW coordinator make a RTW implementation plan, describing who is responsible for implementation of each selected solution, including how this is going to be done and a time path. In addition, the RTW coordinator underlines the importance of own initiative of the worker to achieve RTW; i.e. while the contracted vocational rehabilitation agency is searching for a suitable temporary workplace, the worker himself/herself has the responsibility to look also for a suitable workplace based on the formulated work(place) profile. Next, the RTW coordinator makes a report in which the main items of the participatory RTW process are described: a summary of the prioritised obstacles for RTW, the consensus based solutions, and if possible a concrete work(place) profile. This report is then sent to the sick-listed worker, the labour expert, and the insurance physician. Finally, the RTW coordinator informs the case-manager of the contracted vocational rehabilitation agency.

### Placement in a matching (therapeutic) workplace

Within two days after the brainstorm session the vocational rehabilitation agency is contracted by the RTW coordinator, who sends the formulated work(place) profile to the case-manager of the agency. Within four weeks after this initial contact, the vocational rehabilitation agency has to offer at least two therapeutic workplaces matching with the worker's functional limitations. Next, the sick-listed worker chooses one of these temporary workplaces.

Placement in a temporary workplace is for a maximum of three months with ongoing supportive benefit by the SSA. If required, the case-manager of the vocational rehabilitation agency visits the workplace to instruct and advise/support the worker. And, if necessary, the case-manager of the agency advises the supervisor at the workplace as to how to guide the worker in his/her new work situation.

### Evaluation

Six weeks after the brainstorm session, the RTW coordinator contacts the worker and the case-manager of the vocational rehabilitation agency by telephone to inform whether placement in a temporary workplace has been successful and if everything is satisfactory. If there is still no placement in a temporary workplace, the RTW coordinator contacts the case-manager of the vocational rehabilitation agency to enquire whether and/or when this will be achieved, but stimulates also the worker to find suitable work himself/herself in the mean time. Next, the RTW coordinator makes a final report, describing the process and the outcome of the RTW implementation plan and assigns further guidance to the insurance physician. The case-manager of the vocational rehabilitation agency is asked to make: 1. a report after the intake with the worker, including a description of the temporary workplaces offered to the worker, 2. a mid-term evaluation report six weeks after placement in a temporary workplace and 3. an end evaluation three months after placement in a temporary workplace. With these reports the case-manager of the agency informs the RTW coordinator, the labour expert and the insurance physician about the progress and (end) result of the placement in temporary (therapeutic) work; i.e. contribution to achieve a sustainable RTW.

### Training of the OHC professionals

Instruction and coaching sessions are held for all involved OHC professionals, i.e. insurance physicians and labour experts of the SSA. They also receive a syllabus with detailed information about the participatory RTW program, the protocol, practical summaries and schemes, and practice material. The RTW coordinators receive an additional training, including a role playing and a practise with anonymous cases and reporting. All professionals are offered personal guidance with the first cases to facilitate applying the program. Next, two follow-up session are held with the professionals to discuss difficulties with working with the program and to practise with cases. Finally, to guarantee that the participatory program is carried out according to the required time-path, each SSA front office forms at least two 'participatory RTW program' teams, i.e. 'STEP-UP teams', consisting of an insurance physician, a labour expert and a RTW coordinator.

### Outcomes

#### Effect evaluation

The primary outcome measure in this study is: duration of the sickness benefit period from the first day of reporting sick until ending of the sickness benefit. Recurrence of sickness absence with an accepted sickness benefit claim within 4 weeks after ending of the previous sickness benefit is considered as belonging to the preceding sickness benefit period, on condition that it is due to the same (or related) MSD. Also, for calculation of the total duration of sickness benefit during the one-year follow-up awarded sickness benefit claims are only included when due to same (or related) MSD.

Secondary outcome measures are:

#### - RTW

When measuring the effect of a RTW intervention, it can be expected to take actual RTW as an important outcome measure. For sick-listed employees full RTW and ending of the sickness absence period coincides, in principle. However, for the sick-listed temporary agency worker and the sick-listed unemployed worker moving from being sick-listed to end of sickness benefit does not automatically also mean full RTW. Because in most cases these workers have no workplace/employer to return to, the worker can report being fully recovered from illness or the insurance physician of the SSA can establish full recovery of functional limitations (assessed with regard to last/previous work) without actual RTW of the worker. Therefore, RTW is measured as a separate outcome measure. RTW is defined as: duration from the first day of reporting sick until actual first RTW in any type of paid work or work resumption with ongoing benefits. Since for the majority of these workers there is no workplace to return to, working in the same or different type of work(place) is classified as RTW.

#### - Total number of days of sickness benefit

The total number of days of sickness benefit will be measured for the whole one-year follow-up period. For calculation of the total duration of sickness benefit awarded sickness benefit claims are only included when due to same (or related) MSD.

#### - Severity of MSD

Severity and changes in MSD are measured with the Dutch version of the Nordic Musculoskeletal Questionnaire (DMQ)[36]. In addition, musculoskeletal pain intensity is measured using the Von Korff[37].

#### - Functional status

Functional status, i.e. perceived functional impairments in daily life, is measured using the Dutch translation of the RAND-36[38,39].

#### - General health

General health is measured using the Dutch translation of the RAND-36. Quality of life is measured using the Dutch translation of the Euroquol questionnaire[40].

#### - Coping

Pain coping is measured using the Pain Coping Inventory Scale (PCI)[41].

#### - Attitude, Social Influence and self-Efficacy (ASE) determinants

In line with the development of a participatory RTW program for sick-listed employees with common mental disorders (CMD)[42], the Attitude-Social influence-self-Efficacy (ASE) model was chosen as underlying theoretical framework [43-45] for the development of the new participatory RTW program for sick-listed temporary agency workers and sick-listed unemployed workers. For the developed RTW intervention for CMDs questions about attitude, social influence, self-efficacy, barriers and facilitators were formulated and measured on bipolar five-point Likert scales[46]. This questionnaire is also used in this study.

#### - Direct and indirect costs

Direct costs are paid by the SSA for interventions regarding vocational rehabilitation support, e.g. training/education, for interventions aimed at health promotion, e.g. physical therapy (graded activity) and/or psychological help, or interventions aimed at RTW, e.g. searching for a (temporary) workplace by contracting a vocational rehabilitation agency or temporary placement in work with a willing employer and with an ongoing benefit. Information regarding direct costs is collected from the SSA database and the worker's files after one year of follow-up.

Indirect costs are related to costs due to paid sickness benefit for the sick-listed workers with MSD. When looking at sick-listed temporary agency workers and sick-listed unemployed workers, loss of productivity is not part of the indirect costs. When reporting sick the temporary agency workers immediately falls under the SSA for substituted income, i.e. the sickness benefit. The temporary agency replaces the sick-listed temporary agency worker with a healthy worker at the company/workplace concerned with no productivity loss as a result. Therefore, indirect costs with regard to sick-listed temporary agency workers are the sickness benefit costs paid by the SSA. However, this does not apply to unemployed workers. These workers have no work(place), i.e. there is no productivity. As a consequence, being sick-listed does not result in a productivity loss. Another important fact is that an unemployed worker receives an unemployment benefit. After reporting sick with acceptance of the sickness benefit claim by the SSA, the sick-listed unemployed worker receives a sickness benefit instead of an unemployment benefit. However, the amount of these benefits can differ as this is established using different income conditions. As a result, the sickness benefit can be more than the unemployment benefit. From this perspective, the additional benefit costs are considered indirect costs in this study. Data on paid benefits are collected from the SSA database after the one-year follow-up. Cost-benefit evaluation of the new RTW program is part of this study and will be discussed below.

An overview of the outcome measures and the measurement instruments used, including a time path for all measurements, is presented in table 2.

**Table 2:** Overview of measurements and time path

Measurement	Time path				
	**Baseline**	**3 months**	**6 months**	**9 months**	**12 months**
	**(T0)**	**(T1)**	**(T2)**	**(T3)**	**(T4)**

*Prognostic measures*					
- Demographic variables (e.g. age, gender)	X				
- Last work (function, hours)	X				
- Work status before reporting sick	X				

					

*Primary outcome measure*					
- Duration of sickness benefit	X	X	X	X	X

					

*Secondary outcome measures*					
- RTW	X	X	X	X	X
- Total number of days of sickness benefit	X	X	X	X	X
- Severity of complaints (DMQ)	X	X	X		X
- Pain intensity (Von Korff)	X	X	X		X
- Functional status (RAND-36)	X	X	X		X
- General health (RAND-36)	X	X	X		X
- Quality of life (Euroqol EQ-5D)	X	X	X		X
- Coping (PCI)	X				
- ASE determinants (ASE questionnaire)	X	X			
- Direct and indirect costs					X
- Patient satisfaction (PSOHSQ)*		X			

* patient satisfaction with occupational health services is only measured in the intervention group (as part of the process evaluation)

### Data collection

Most outcome variables are measured using self-report questionnaires. At the intake appointment with the research assistant, after informed consent, the sick-listed worker fills in the baseline questionnaire. All participants are followed one year with measurements, i.e. questionnaires, at 3, 6, 9 and 12 months after baseline. These questionnaires are sent by postal mail. If the received questionnaire is incomplete or if anything is unclear, the researcher or research assistant contacts the participant to clarify and, if possible, to complete the questionnaire. Data on sickness benefit are registered by the SSA and are acquired from the SSA database after one-year follow-up. These data are checked with information regarding sickness benefit as registered by the insurance physician of the SSA in the medical file of the sick-listed worker, and the self-reported information in the questionnaires. Data regarding diagnosis and occupational health care interventions are obtained from the SSA database and medical file of the worker at the SSA. Data regarding RTW are obtained from both the SSA database, including the workers' file, and the self-report questionnaires.

### Prognostic measures

At baseline information is gathered regarding demographic variables, such as gender, age, and level of education. Also, information regarding last work, e.g. type of previous work and number of working hours, and the work status (working or not working) directly prior to the baseline measurement is collected. This is partly based on findings in the international literature [47-49], indicating that the work status before sickness absence is a prognostic factor for the duration of sick leave and work disability.

### Cost-benefit evaluation

Cost-benefit is evaluated from the insurer's perspective. Direct and indirect costs are measured with data from the SSA database and the worker's files, as mentioned above. Direct costs are calculated from the amount of paid occupational health care interventions by the SSA. Indirect costs are calculated from the (additional) costs of paid sickness benefit.

### Process evaluation

After implementation a process evaluation is conducted among the participants in the intervention group. Three months after inclusion a questionnaire is sent to the worker, the insurance physician, the labour expert, the RTW coordinator and the case-manager of the contracted vocational rehabilitation agency. For the participants, the process evaluation questions are included in the 3-months questionnaire and sent by postal mail. Questions are asked regarding applicability, compliance, satisfaction and barriers regarding (implementation of) the new RTW program in practice. Patient satisfaction is measured using the Patient Satisfaction with Occupational Health Services Questionnaire (PSOHQ)[50]. In addition, when all participants in the intervention group have had the opportunity to receive the new RTW program, i.e. 3 months after inclusion of the last participant, focus group meetings are held among the staff, management and involved occupational health care professionals of the SSA, and the case-managers of the vocational rehabilitation agencies concerned. The content of these focus groups are based on the principles of context-analysis as proposed by Grol and Wensing[29].

Finally, standardised schemes are used to collect data regarding the identified barriers for RTW, the formulated solutions and the resulting consensus-based RTW implementation plan. The collected data will be analysed qualitatively and quantitatively. In addition, the identified barriers and solutions will be classified using the Ergonomic Abstracts scheme[51,52].

### Sample size

In this study the primary outcome measure is duration of the sickness benefit period. Recurrence of sickness absence (due to the same or related MSD) with an accepted sickness benefit claim within 4 weeks after ending of the previous sickness benefit is considered as belonging to the preceding sickness benefit period. As a starting-point for calculating the sample size we assume that a Hazard Ratio (HR) of 2.0 is the minimal clinical and societal relevant ratio, indicating that temporary agency workers and unemployed workers in the intervention group end their sickness benefit period twice as quickly compared to the workers in the control group. This HR is based on comparable studies on sickness absence and RTW of short-term sick-listed employees[25,46,53-55]. Assuming that the sickness benefit will end for 2/3 of the participants during the one-year follow-up period, and based on a power of (1-β =) 0.80 and a two-sided significance level of 0.05 (α) a sample size of 100 participants (n = 2 × 50) is needed[56]. Since there is a continuous registration of sickness benefit duration by the SSA, a high loss to follow-up with regard to the primary outcome is not expected. However, based on comparable research[57,58] loss to follow-up of 10% is taken into account. This results in 110 participants (n = 2 × 55) to be included in the study. Next, potential clustering of cases assessed by the same insurance physician is taken into account, since the insurance physician plays a key role in acceptance of the sickness benefit claim and the assessment of (sufficient) recovery of functional limitations. For this calculation an ICC of 0.05[25,46] is used and a minimal number of clusters of 10 (i.e. 5 front offices with at least 2 participating insurance physicians at each office). This results in 160 participants (n = 2 × 80) to be enrolled in the study.

### Randomisation procedure

An independent statistician performs the randomisation, using computer-generated randomisation tables. To prevent unequal distribution of relevant prognostic baseline characteristics, before randomisation the sick-listed workers are pre-stratified based on two important prognostic factors, namely type of worker [47-49], i.e. temporary agency worker or unemployed worker, and degree of mental or physical work demands (light or heavy) in last work before current sickness absence[59,60]. Next, block randomisation (using blocks of four allocations) is applied to ensure equal group sizes within each stratum. A separate block randomisation table is generated for each of the five participating front offices of the SSA. Next, the researcher prepares for each stratum opaque sealed envelopes, containing either a referral to the new RTW program group or to the usual care group. After informed consent and completing the baseline questionnaire, the temporary agency worker or unemployed worker is asked to choose one of the two succeeding envelopes of the correct stratum. Then, the worker is asked to open the envelope and write down his/her name and date on the note with the randomisation result.

### Blinding

Since the occupational health care professionals can be involved in guidance of participants of both the intervention group and the usual care group and because the new RTW program contains several new elements compared to usual care, i.e. a combined consult with the insurance physician and the labour expert, meetings with the RTW coordinator, and contracting of a vocational rehabilitation agency for finding a temporary (therapeutic) workplace, the occupational health care professionals cannot be blinded for the allocation. Furthermore, most outcome measures are self-reported, which also makes blinding for the participants not possible. However, the occupational health care professionals and RTW coordinators are not involved in the assessment of the outcomes. Moreover, since all follow-up questionnaires are sent to the participants by postal mail, it is unlikely that direct influence of the researchers or occupational health care professionals will occur.

Since the registration of sickness benefit is done by the SSA, these measurements can be derived from the computerised SSA database. Therefore, bias due to a lack of blinding is prevented for this outcome. Blinding for the secondary outcomes is not possible, because these measurements are derived from self-reported data. After randomisation all participants receive a research code consisting of a unique consecutive number. All data will be put in the computer by a research assistant, using this research code, to guarantee that analyses of the data by the researcher will be blinded.

### Co-interventions and compliance

Unfortunately, in this pragmatic RCT it is not possible to avoid co-interventions during the intervention period, because asking the temporary agency workers, the unemployed workers, and the occupational health care professionals of the SSA to stop or not start with other treatments will lead to less participation. To measure the compliance with the new RTW program, the participants, the occupational health care professionals, the RTW coordinators, and the case-managers of the vocational rehabilitation agencies are asked independently about all interventions applied. Also, both the intervention group and the control group are asked about co-interventions in each follow-up questionnaire. If necessary, we can adjust for co-interventions in the multivariate analysis.

### Contamination

Since randomisation takes place at the workers level, the insurance physicians, the labour experts, and the RTW coordinators who are trained in the new RTW program can also be involved in RTW guidance of a sick-listed worker in the usual care group. Therefore, the occupational health care professionals are asked to avoid the use of (components of) the RTW program in the guidance of participants in the usual care group.

### Statistical analyses

All statistical analyses will be performed at worker's level and according to the intention-to-treat principle, i.e. participants will remain in the group (intervention group or control group) to which they were allocated after randomisation at baseline. To check the success of the randomization procedure descriptive statistics will be used, comparing the baseline measurements of both groups. If necessary, analyses will be adjusted for prognostic dissimilarities. To asses the presence of bias due to protocol deviations, the results of the intention-to-treat-analyses will be compared to per-protocol analyses, including only those participants who were treated according to the intervention protocol.

#### Effect evaluation

In this study survival analysis will be used to analyse sickness benefit data with regard to the first period of sickness benefit. To describe the duration until ending of sickness benefit in both groups, the Kaplan Meier method will be used. In order to calculate hazard ratios the Cox proportional hazard model will be applied. If necessary, standard errors will be corrected for clustering. Differences in total days of sickness benefit during the one-year follow-up will be analysed with a general linear model. If necessary, the results will be adjusted for dissimilarities at baseline. Longitudinal random coefficient analyses will be used to assess differences in secondary outcome measures. Finally, intraclass correlation coefficients will be calculated at the level of the insurance physician, since the insurance physician plays a key role in acceptance of the sickness benefit claim and the assessment of (sufficient) recovery of functional limitations, i.e work ability, with ending of the sickness benefit.

#### Cost-benefit evaluation

Direct and indirect costs from the insurer's perspective will be calculated for each individual participant. Bootstrapping will be used for pair wise comparing of the group means to calculate mean differences in direct, indirect and total costs between both groups of workers. Confidence intervals (95%) will be computed by bias corrected and accelerated bootstrapping. The mean net monetary benefit (NMB) of the new RTW program compared to usual care will be calculated.

## Discussion

This study focuses on a vulnerable group within the working population, namely temporary agency workers and unemployed workers, sick-listed due to MSD. For this group of workers a new participatory RTW program has been developed[16] aimed at making a consensus-based RTW implementation plan, realising structural communication among important stakeholders involved in vocational rehabilitation of the sick-listed worker, and offering the possibility of a (therapeutic) workplace to RTW. This paper describes the design of a randomised controlled trial to investigate the effectiveness, the cost-benefit and feasibility of this new RTW program.

### Strengths of the study

Strength of this study is the focus on a vulnerable group within the working population, i.e. workers without an employment contract or with a flexible labour arrangement. These workers have a greater distance to the labour market and an increased risk for (long-term) work disability. This is reflected in the absenteeism and RTW patterns[17]. For these workers there can be gained a lot by efforts that aim at improving occupational health care and by minimising the 'labour market handicap', i.e. creating an actual RTW perspective to reduce short- and long-term sickness absence and work disability[13,17].

Another strength of this study is the data collection from the SSA database. Duration of the sickness benefit period after the first day of reporting sick is the primary outcome measure in this study. Registration of awarded sickness benefit by the SSA provides reliable data because of socio-political and financial interests. In the Netherlands sickness benefit is paid from public means. Therefore the performance of the SSA is monitored by the Inspection Service for Work and Income on behalf of the Dutch Ministry of Social Affairs and Employment. As a result, loss of data of the primary outcome due to loss to follow-up is limited. However, because after ending of the sickness benefit the SSA has, in many cases, no longer data on RTW, data collection from the SSA database alone might underestimate RTW during the one-year follow-up. Therefore data on RTW are collected from both the self-report questionnaires and the SSA database.

A third strength of this study is that it includes a feasibility study. To gain more insight in the potential benefits, applicability and barriers of the new RTW program in daily practice. And, if possible, to identify elements of the RTW program that contribute to the effect.

### Limitations of the study

A limitation in this study is the absence of blinding of both the sick-listed workers and the occupational health care professionals of the SSA for allocation to the usual care group or intervention group. However, this is not possible due to the nature of the participatory intervention program.

Secondly, because the insurance physician of the SSA has no role in the inclusion of participants, a limitation of this study is the possibility of bias due to self-selection of workers. On the other hand, introduction of bias due to selection of participants by the insurance physician is limited, since the selection procedure is done by the researchers using strict inclusion and exclusion criteria.

A third limitation is the fact that generalizing the results of this study to another context, e.g. other countries, should be done with caution. The new RTW program is specifically tailored to the Dutch context using the Intervention Mapping process [26-28]. Application of this intervention in a different setting should be preceded by tailoring of the program, taking into account the specific characteristics of the social, political and cultural context[26-29,61] in which the program will be implemented and used.

### Impact of study findings

Flexible labour market arrangements have expanded enormously over the last decades [62-64]. However, workers with non-standard labour arrangements represent a vulnerable group within the working population. As mentioned earlier, these workers experience more health problems, have an increased risk for work disability[10,14,15], and access to vocational rehabilitation interventions [18-20] is in many countries not available or only limited for these workers. More should be done for them to achieve a sustainable contribution to the labour force. In addition, given the international trend of an ageing workforce, there is a need for active labour-market policies[65]. From this perspective, it is not only important to improve participation of older workers[65,66], but to also utilise and strengthen present and potential vulnerable labour force sources. In line with this, more (tailor-made) RTW interventions should be aimed at the group of flexible workers, including workers without an employment contract. The results of this RCT can contribute to this need for tailor-made occupational health care.

Secondly, the absence of a workplace to return to when sick-listed has been identified as a major obstacle for these workers to successful (re-)enter the labour market[16,17]. Creating an actual RTW perspective can have a considerable impact. Positive results in this study may lead to implementation of the program in usual care in the Netherlands. In addition, this study is aimed at workers without an employment contract, sick-listed due to MSD. Results may offer perspective for the development of participatory RTW interventions for these workers, sick-listed with other health problems, e.g. common mental disorders.

Furthermore, sickness absence is considered a major public health and economic problem. The involved costs are enormous with a disproportionate contribution by long term work disability. Long term sickness absence can contribute up to 75% of absence costs[67]. In the Netherlands, the participatory RTW program already proved to be successful for sick-listed employees with low back pain with an average reduction of sickness absence of 27 days[25]. If a comparable reduction of sickness absence, i.e. duration of sickness benefit, can be achieved in this study, the benefits for the Dutch Social Security System will be substantial. Finally, during the development of the participatory RTW program it became evident that there is a need for more uniformity with application of evidence-based interventions in occupational health care by the SSA[16]. The occupational health care professionals at the SSA can benefit from a structured approach to identify and discuss barriers for RTW and making of a consensus-based RTW action plan.

Results of this study will become available in 2010.

## Competing interests

The authors declare that they have no competing interests.

## Authors' contributions

SJV is responsible for the data collection and drafted the manuscript. SJV, JRA and AJMS developed the study design and intervention protocols. SJV and JRA are responsible for the general coordination of the study. All authors have read and corrected draft versions of the manuscript and approved the final manuscript.

## Pre-publication history

The pre-publication history for this paper can be accessed here:

http://www.biomedcentral.com/1471-2474/11/60/prepub
